# The sponsor-proxy model: Bridging basic science discovery and investigator-initiated clinical trials

**DOI:** 10.1017/cts.2026.10782

**Published:** 2026-06-24

**Authors:** Xin Liu, Doris Shank, Junxuan Lü, Monika Joshi

**Affiliations:** 1 Penn State Cancer Institute, Pennsylvania State University, Hershey, PA, 17033, USA; 2 Department of Neuroscience and Experimental Therapeutics, Penn State College of Medicine, Hershey, PA, 17033, USA; 3 Center for Cannabis and Natural Product Pharmaceutics, Pennsylvania State University College of Medicine, Hershey, PA, 17033, USA; 4 Department of Medicine, Division of Hematology and Oncology, Penn State College of Medicine, Hershey, PA, 17033, USA

**Keywords:** investigator-initiated trials, translational science, clinical trial infrastructure, regulatory science, investigational new drug

## Abstract

Translating academic discoveries into clinical investigation remains a major barrier in translational science, particularly when investigators lack the regulatory infrastructure required to sponsor clinical trials. These challenges are especially pronounced for natural product therapeutics, which often lack commercial sponsorship despite promising preclinical evidence. The Penn State Cancer Institute established an Investigator-Initiated Trial Sponsor Support Unit (SSU) to facilitate investigator-initiated trials and support progression toward National Cancer Institute designation. We describe a translational science case study of a Sponsor-Proxy operational model in which a centralized institutional unit performs sponsor-level regulatory and trial infrastructure functions while investigators retain scientific and clinical leadership. Two National Institutes of Health-funded trials evaluating Angelica gigas Nakai extract (INM176) illustrate this framework. The SSU authored regulatory documentation including Investigator’s Brochures, coordinated Investigational New Drug submissions, and supported trial infrastructure while clinical teams maintained participant oversight. Both trials achieved activation timelines of 112 and 105 days, shorter than previously reported activation times and within National Cancer Institute operational benchmarks. Sponsor-level oversight continued throughout trial conduct, including regulatory reporting, safety monitoring, and protocol amendment support. This case study demonstrates how centralized institutional expertise can overcome sponsor-level barriers and enable translation of basic science discoveries into investigator-initiated early-phase clinical trials.

## Introduction

Investigator-initiated trials (IITs) play a critical role in translating discoveries from academic laboratories into clinical testing. However, many promising scientific findings fail to reach human testing because investigators lack the regulatory infrastructure required to serve as clinical trial sponsors. These challenges are particularly pronounced for natural product therapeutics, which often demonstrate compelling preclinical activity but receive limited commercial investment and therefore rely on investigator-initiated translational pathways [1]. Regulatory development of botanical therapeutics is further complicated by the heterogeneous nature of these products, which often contain multiple active constituents and require extensive characterization of raw materials, manufacturing processes, and batch consistency to support Investigational New Drug (IND) submissions [2].

The Penn State Cancer Institute (PSCI) serves a 28-county catchment area in central Pennsylvania comprising approximately four million residents, many of whom live in rural Appalachian communities where geographic distance and socioeconomic factors create barriers to healthcare access and participation in clinical research [1,3]. Expanding locally driven translational research was therefore both a scientific and public health priority. These geographic characteristics also highlight the importance of operational approaches that enable clinical research participation without requiring frequent travel to a central academic medical center.

In 2018, PSCI established an Investigator-Initiated Trial Sponsor Support Unit (IIT SSU) as part of institutional development aligned with Cancer Center Support Grant objectives supporting progression toward National Cancer Institute designation. Prior to this initiative, investigator-initiated translational trials were uncommon due to limited sponsor-level expertise and fragmented operational support. The program aimed to mentor investigators, ensure regulatory compliance, and promote development of internally generated (“homegrown”) clinical trials. Most IITs conducted at PSCI are early-phase studies, primarily Phase I and Phase II trials, which represent an essential foundation for therapeutic development and early clinical evaluation of emerging cancer treatments.

Translation of promising academic discoveries into IITs also requires investigators to assume complex sponsor responsibilities, including preparation of regulatory submissions, development of trial documentation such as protocols and Investigator’s Brochures (IBs), management of Trial Master Files (TMFs), safety reporting, and ongoing regulatory communication with the U.S. Food and Drug Administration (FDA) [4,5]. These activities require specialized regulatory knowledge and operational coordination that are rarely available to individual investigators, particularly basic scientists seeking to translate laboratory discoveries into clinical investigation.

To address these barriers, PSCI developed a centralized operational framework in which institutional expertise performs sponsor-level regulatory and trial infrastructure functions while investigators retain scientific and clinical leadership. This framework, referred to here as the Sponsor-Proxy model, allows centralized operational teams to execute the sponsor-level regulatory and operational responsibilities while maintaining investigator-driven scientific oversight.

**Figure 1.**
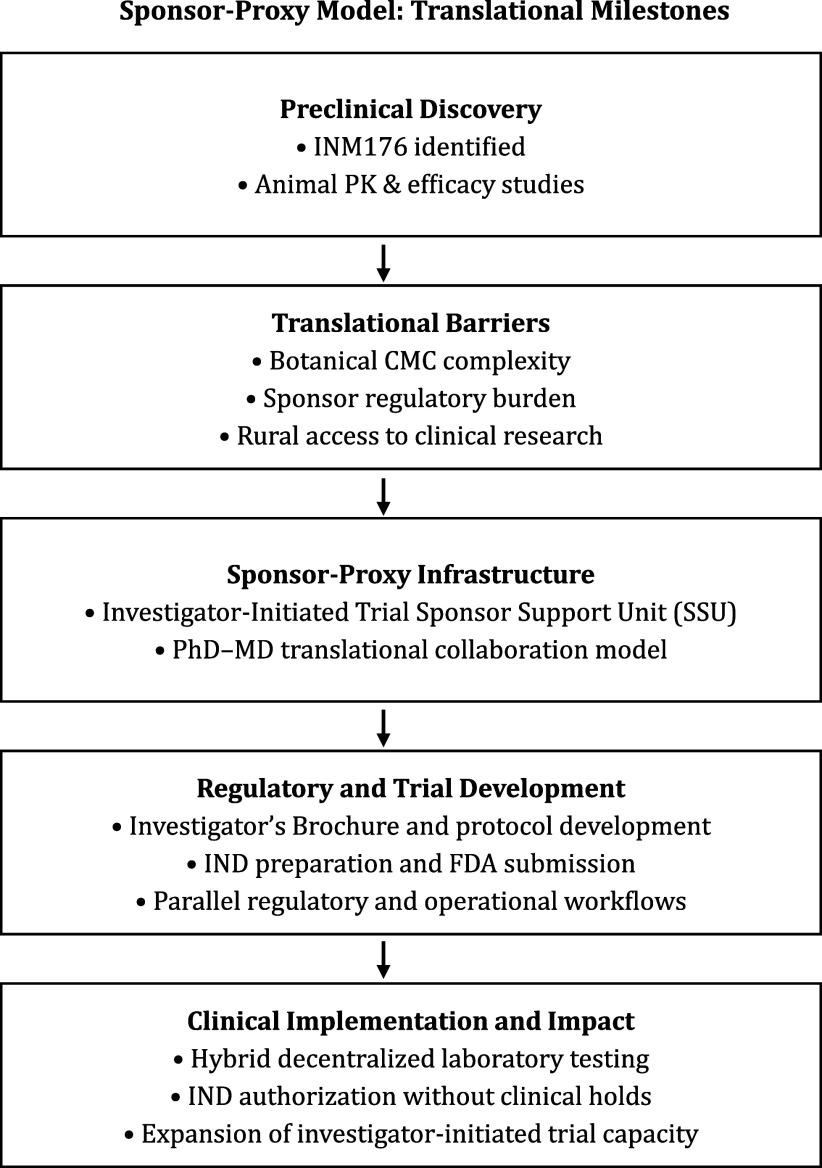
Figure 1 long description. Translational milestones enabled by the Sponsor-Proxy model. The figure illustrates the progression from preclinical discovery of *Angelica gigas Nakai* extract (INM176) through regulatory development, trial activation, and clinical implementation. The Sponsor Support Unit (SSU) enables this progression by integrating sponsor-level regulatory, operational, and clinical trial infrastructure, addressing key translational barriers and facilitating investigator-initiated clinical trials.

A defining feature of the IIT SSU program is its capacity to translate discoveries generated by basic science investigators into early-phase clinical studies. By assuming sponsor-level operational responsibilities, the Sponsor-Proxy model bridges the gap between laboratory discovery and clinical implementation, enabling PhD-led innovations to advance to investigator-initiated clinical trials (Figure 1).

*Angelica gigas* Nakai extract (INM176), whose chemical structure has been previously characterized [6], provides an illustrative example of this translational pathway.

## Approach: the sponsor-proxy model

1. **Conceptual Framework**

**Figure 2.**
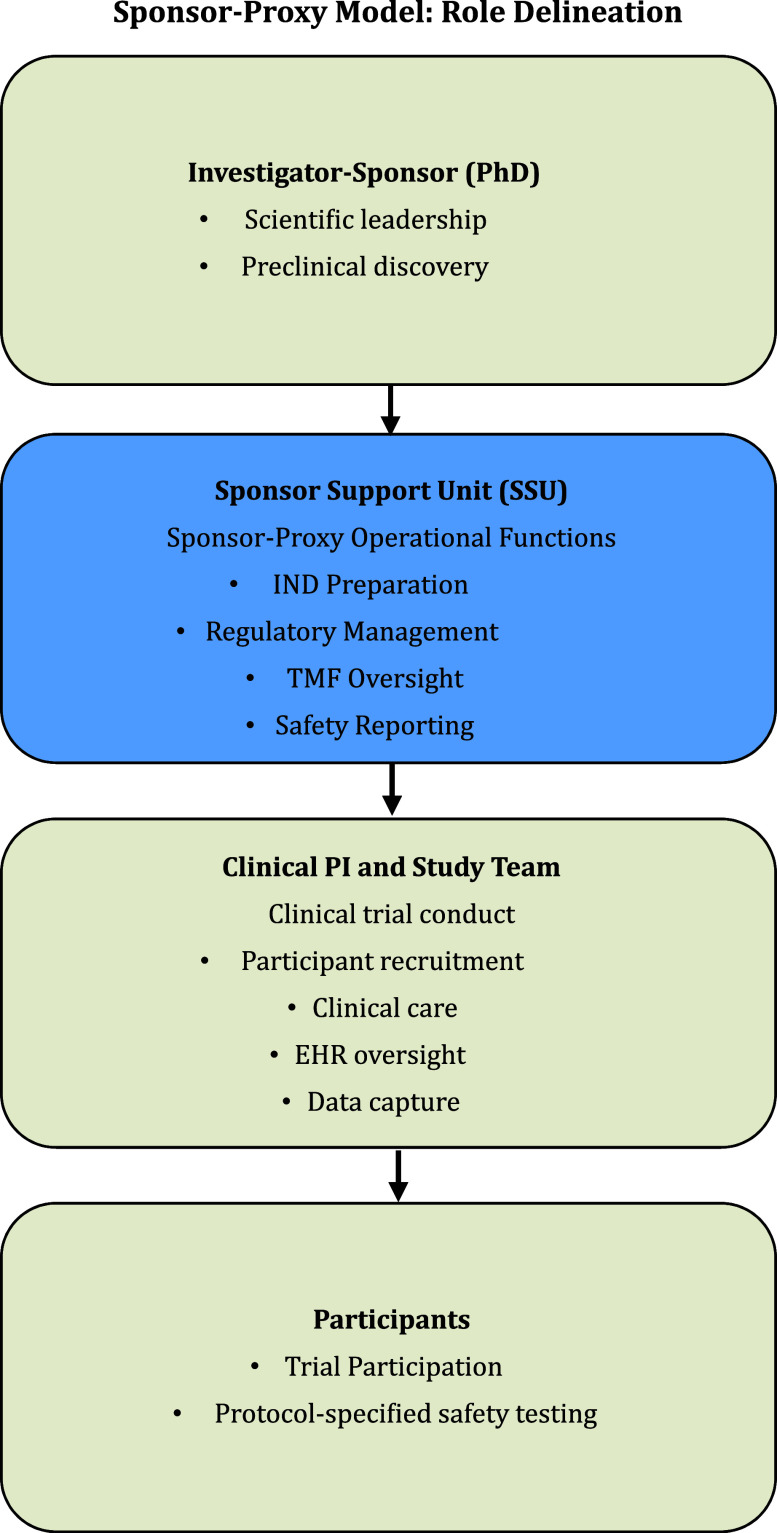
Figure 2 long description. Role delineation in the Sponsor-Proxy operational model. The Sponsor-Proxy model delineates scientific sponsorship, sponsor-level regulatory and operational responsibilities, and participant-level clinical oversight. The Sponsor Support Unit (SSU) performs centralized regulatory and operational functions on behalf of the sponsor, while the clinical principal investigator (PI) and study team maintain direct clinical oversight of trial participants.

Under the Sponsor-Proxy model, the academic investigator remains the regulatory sponsor, while the Sponsor Support Unit (SSU) performs centralized sponsor-level regulatory and operational functions on behalf of the sponsor (Figure 2). Scientific leadership remains investigator-driven, while clinical oversight resides with the physician principal investigator (PI) and study team responsible for participant care and trial conduct. This structure separates scientific leadership, sponsor-level regulatory operations, and participant-level clinical oversight into distinct but coordinated domains.

1. **SSU Expertise and Workforce Model**

The SSU is staffed by highly trained clinical research professionals holding either master’s- or doctoral-level degrees and possessing extensive experience in clinical trial development and execution. Team members perform integrated sponsor-level functions, including regulatory submissions, protocol and Investigator’s Brochure development, continuous trial oversight, and training of study personnel. This cross-functional model enables staff to contribute across the full trial lifecycle, ensuring operational continuity and consistent regulatory standards without reliance on multiple specialized roles.

1. **Translational Integration**

Basic science investigators often possess deep mechanistic insight but limited experience with regulatory requirements for clinical trial sponsorship. Within the Sponsor-Proxy framework, SSU personnel translated preclinical toxicology and pharmacokinetic findings into clinical safety monitoring plans, dose-escalation strategies, and IND-ready regulatory documentation, enabling laboratory discoveries to be operationalized as early-phase clinical studies.

1. **Operational Workflow**

The SSU currently supports a growing portfolio of investigator-initiated trials, including two NIH-funded INM176 studies, through centralized sponsor-level activities performed on behalf of the sponsor, including:

- protocol and informed consent development and amendment.
- Investigator’s Brochure preparation and maintenance.
- Integration of chemistry, manufacturing, and controls (CMC) documentation.
- IND preparation, submission, and regulatory maintenance.
- Trial Master File oversight and maintenance.
- Monitoring plan development.
- Coordination of FDA and Data and Safety Monitoring Committee (DSMC) reporting.
- Coordination of sponsor–investigator communications and documentation.

The clinical PI team retained responsibility for participant recruitment, clinical care, real-time electronic health record (EHR) oversight, and clinical data capture and management. The division of responsibilities between the SSU and the clinical PI study team across the clinical trial lifecycle is summarized in Table 1.

**Table 1.** Regulatory and translational barriers encountered during natural product IND development Table 1 long description.

Regulatory domain	Example FDA information request	Sponsor-proxy operational response
IND documentation	Investigator’s Brochure requested during IND review	SSU collaborated with the scientific sponsor to synthesize preclinical pharmacology, toxicology, and prior clinical data into a formal Investigator’s Brochure
Botanical product characterization	Clarification of botanical extract composition and active compound content	Coordinated manufacturer documentation and independent analytical verification of decursin content
Chemistry, Manufacturing, and Controls (CMC)	Documentation of manufacturing processes, technology transfer, and contaminant testing	Integrated manufacturing records, certificates of analysis, and quality testing into FDA-compliant CMC documents
Clinical protocol safety design	Revision of dose-limiting toxicity definitions, eligibility criteria, and safety monitoring procedures	Coordinated protocol revisions, statistical design updates, and integration of FDA safety guidance
Human subject protections	Expansion of informed consent language including addressing adverse events and reproductive risk	Updated informed consent documentation to align with regulatory requirements

Clinical research procedures for pharmacokinetic study, including blood collection, vital sign assessment, and electrocardiogram (EKG) monitoring, were conducted with support from institutional Clinical and Translational Science Institute (CTSI)-supported clinical research center (CRC) resources.

1. **Hybrid-Decentralized Access**

Safety laboratory assessments could be completed locally within the Penn State Health network up to five days before scheduled visits. This approach reduced participant travel burden while maintaining centralized clinical oversight by the study team.

## Barriers to translation and mitigation

Several translational barriers were addressed through the Sponsor-Proxy operational framework:

1. **Natural Product Regulatory Complexity**

Botanical products require detailed documentation of extract composition, manufacturing processes, and standardization of active compounds. The SSU translated manufacturing and quality-control data into FDA-compliant regulatory documentation.

1. **Sponsor Experience Gap**

Basic science investigators often lack experience with IND regulatory requirements. Centralized SSU expertise provided operational sponsor support while allowing investigators to retain scientific leadership.

1. **Rural Access Constraints**

Geographic dispersion within the PSCI catchment area can limit clinical trial participation. Local laboratory testing within the Penn State Health network reduced participant travel burden while preserving centralized clinical oversight.

1. **Trial Start-up Inefficiencies**

Coordinated parallel workflows between regulatory preparation, protocol development, and operational planning accelerated trial activation timelines.

## Translational implementation and outcomes

The following observations illustrate how the Sponsor-Proxy model functioned in real-world IND development and trial activation.

1. **IND Development and Regulatory Barriers**

Implementation of the Sponsor-Proxy model supported two investigator-initiated trials evaluating *Angelica gigas Nakai* extract (INM176). During IND review, several regulatory information requests were received that reflect common challenges in natural product clinical development. These included clarification of botanical extract composition, manufacturing processes, and documentation of CMC data, which are recognized challenges in botanical drug development [2]. FDA reviewers also requested clarification regarding investigational product formulation relative to commercially available supplements.

Regulatory review of the pharmacokinetic trial also identified gaps in standard IND documentation commonly required for early-phase investigator-initiated studies. The FDA requested submission of an Investigator’s Brochure summarizing preclinical pharmacology, toxicology, and prior human safety data for the investigational botanical product. These requests highlight the operational complexity associated with investigator-initiated IND development for botanical products.

Key regulatory and translational challenges encountered during natural product IND development, along with corresponding Sponsor-Proxy operational responses, are summarized in Table 2.

**Table 2.** Operational responsibilities in the sponsor-proxy model Table 2 long description.

Operational domain	Sponsor Support Unit (SSU)	Clinical PI study team
Trial design	Protocol development, Investigator’s Brochure preparation	Clinical feasibility input
Regulatory oversight	IND preparation and submission, FDA communication	Site regulatory compliance
Documentation	Trial Master File management	Source documentation
Safety oversight	Sponsor safety reporting, DSMC coordination	Clinical safety monitoring
Monitoring	Monitoring plan development	Site coordination
Data management	Oversight of sponsor documentation	Data capture and entry
Participant management	—	Recruitment, clinical care, EHR monitoring

1. **Sponsor-Proxy Operational Responses**

The SSU coordinated responses to regulatory information requests by working with the product manufacturer to obtain manufacturing documentation and certificates of analysis, integrating analytical verification of active compound content, and translating technical manufacturing data into FDA-compliant regulatory documentation.

The SSU also collaborated with the sponsor to synthesize available preclinical and clinical evidence into a comprehensive Investigator’s Brochure suitable for regulatory submission.

Additional FDA feedback addressed refinement of clinical protocol elements, including eligibility criteria, definition of dose-limiting toxicities, safety monitoring procedures, and clarification of the dose-escalation framework. The SSU coordinated protocol revisions, statistical design updates, and informed consent modifications to align with FDA guidance and ensure appropriate safety monitoring.

1. **Trial Activation and Operational Outcomes**

Implementation of the Sponsor-Proxy model enabled two investigator-initiated trials:

- Pharmacokinetic study (ClinicalTrials.gov: NCT05375539)
- Ongoing Phase I/II trial (ClinicalTrials.gov: NCT06600698)

Both trials achieved PRC (Protocol Review Committee)-to-activation timelines of 112 and 105 days respectively, reflecting efficient progression from institutional scientific review to trial opening. These timelines compare favorably with previously reported activation times for investigator-initiated oncology trials (∼163–191 days) and with the National Cancer Institute Operational Efficiency Working Group benchmark of 180 days from protocol submission to trial activation [7–10].

IND submissions received FDA authorization without clinical holds.

Centralized operational management allowed investigators to focus on scientific and clinical responsibilities while maintaining regulatory compliance throughout IND review and trial conduct.

Beyond the INM176 studies, the IIT SSU is currently supporting an additional investigator-initiated translational program led by a basic science PhD investigator involving a natural product–based intervention. This study has obtained IND authorization, completed site initiation activities, and has recently opened to accrual, further demonstrating reproducibility of the Sponsor-Proxy framework across independent translational programs.

Hybrid decentralized clinical laboratory workflows further improved feasibility for participants residing in rural areas by allowing safety laboratory testing to occur at local health system facilities while maintaining centralized oversight by the clinical study team.

## Discussion

This translational science case study demonstrates how centralized sponsor-level infrastructure can enable progression of academic discoveries into IND-authorized clinical trials within an academic cancer center where dedicated sponsor infrastructure is limited. A defining achievement of the Investigator-Initiated Trial Sponsor Support Unit (IIT SSU) program was enabling investigator-driven discoveries to advance from preclinical research and early human safety evaluation into disease-specific clinical testing, a translational step that frequently encounters operational barriers in academic settings [1].

The PSCI Clinical Trials Office’s Sponsor Support Unit (SSU) provides a practical framework for operationalizing investigator-initiated trials within an institution where sponsor-level regulatory and operational functions are not distributed across multiple specialized departments. Instead, the SSU integrates sponsor-level responsibilities within a small cross-functional team capable of supporting study design, regulatory execution, and trial implementation. This structure enabled efficient activation of two National Institutes of Health–funded trials evaluating *Angelica gigas* Nakai extract (INM176), achieving PRC-to-activation timelines of 112 and 105 days. These timelines are shorter than previously reported activation durations for investigator-initiated trials, including 163 days reported at Washington University School of Medicine, 171–191 days across U.S. academic and community cancer centers, and approximately 4.2 months reported in a dedicated clinical trials unit [8–10]. These findings also compare favorably with the National Cancer Institute Operational Efficiency Working Group benchmark of 180 days for trial activation [7]. Notably, the trials described in this study were supported by NIH funding, a category historically recognized as less likely to meet benchmark timelines, further underscoring the operational efficiency of the Sponsor-Proxy model [7].

Operational efficiency was supported by highly trained clinical research professionals holding master’s- or doctoral-level degrees and possessing extensive experience in clinical trial development, regulatory submissions, and protocol implementation. This integrated expertise allows staff members to contribute across multiple sponsor-level responsibilities throughout the trial lifecycle, ensuring continuity of regulatory oversight and operational coordination. The program also emphasizes mentorship and hands-on training of additional clinical research professionals with graduate-level expertise to support long-term institutional capacity for investigator-initiated trial sponsorship.

Institutional programs supporting clinical translation have expanded through Clinical and Translational Science Award (CTSA) initiatives, including Regulatory Knowledge and Support cores and collaborative infrastructures such as the Trial Innovation Network [11,12]. At Penn State, these efforts are supported through the NIH-funded CTSI. These programs provide consultation, education, and coordination resources that assist investigators in navigating translational research processes. At Penn State, CTSA-supported initiatives such as the pilot wrap-around support program described by Stoltzfus and colleagues provide enhanced mentoring, project management, and coordination for internally funded translational projects [13]. In addition, Penn State CTSI-supported CRC resources offer dedicated research space and clinical research staff that can facilitate study conduct and reduce burden on clinical care environments. However, these consultative frameworks typically do not assume the sponsor-level regulatory responsibilities required for investigator-initiated IND studies. The Sponsor-Proxy model complements these programs by extending institutional support from advisory consultation to continuous operational execution across the clinical trial lifecycle, including regulatory submission preparation, safety reporting.

A major barrier to clinical translation in academic environments is that basic science investigators who generate promising therapeutic discoveries frequently lack the regulatory and operational experience required to function as investigator-sponsors. While these investigators possess deep mechanistic expertise, IND sponsorship requires preparation of extensive regulatory documentation, management of TMFs, safety reporting, protocol amendment oversight, and ongoing regulatory communication with the FDA [4,5]. These operational responsibilities are rarely part of traditional scientific training and can present substantial obstacles to advancing laboratory discoveries into clinical investigation. By assuming sponsor-level operational responsibilities while investigators retain scientific leadership, the Sponsor-Proxy model enables basic science discoveries to progress into investigator-initiated clinical trials.

The value of this framework is particularly evident in natural product translational research. Botanical therapeutics frequently demonstrate promising biological activity in preclinical studies but encounter substantial barriers to clinical investigation due to regulatory requirements surrounding product characterization, standardization, and manufacturing documentation [11,12], These complexities often exceed the regulatory expertise available to individual academic investigators, particularly when industry sponsorship is absent. By integrating regulatory, operational, and clinical trial expertise within a centralized team, the Sponsor-Proxy framework enables investigators to translate natural product discoveries into regulatory-ready clinical trials despite limited commercial support.

The regulatory review process for the INM176 trials further illustrated practical translational barriers commonly encountered in investigator-initiated IND studies. FDA information requests addressed botanical extract standardization, manufacturing documentation, and refinement of clinical safety monitoring procedures. Development of a formal Investigator’s Brochure during IND review also highlighted a common gap in regulatory documentation preparation when basic science discoveries transition into clinical testing. Through coordination of manufacturing documentation, regulatory submissions, and protocol revisions, the Sponsor-Proxy framework enabled investigators to address these regulatory requirements while maintaining efficient trial activation timelines. These regulatory requests are consistent with FDA guidance on botanical drug development, which emphasizes the need for rigorous characterization of botanical raw materials, manufacturing processes, and chemical constituent profiles to ensure batch consistency and clinical interpretability [2].

Beyond the INM176 studies, the IIT SSU program is currently supporting additional investigator-initiated translational studies led by basic science investigators. One such natural product–based study has obtained IND authorization, completed site initiation activities, and has recently opened to accrual, further demonstrating the reproducibility of the Sponsor-Proxy framework across independent translational studies. These experiences suggest that the Sponsor-Proxy framework is reproducible and adaptable across multiple translational efforts and therapeutic domains. Hybrid decentralized clinical laboratory workflows further demonstrated how integrated health systems can expand research accessibility for underserved populations while maintaining centralized clinical oversight, providing a pragmatic strategy for improving research participation in geographically dispersed regions.

Collectively, these findings suggest that aligning sponsor-level regulatory infrastructure with institutional translational research resources can help overcome common bottlenecks in academic drug development. The Sponsor-Proxy model therefore represents a reproducible institutional strategy for enabling investigator-initiated clinical translation in academic environments where dedicated sponsor infrastructure may be limited.

From a translational science perspective, the Sponsor-Proxy framework illustrates how integrating regulatory execution, operational coordination, and scientific leadership within institutional infrastructure can facilitate advancement of investigator-driven discoveries from laboratory research to early-phase clinical testing. As academic centers increasingly seek to translate innovative therapies into clinical investigation, operational models that bridge scientific discovery and regulatory implementation may play an important role in accelerating investigator-initiated translational research.

Despite these strengths, several limitations should be considered.

## Limitations

This report describes implementation of the Sponsor-Proxy framework within a limited number of investigator-initiated trials at a single academic cancer center. Although the model demonstrated successful regulatory navigation and trial activation in these studies, broader evaluation across additional trials and institutional settings would provide further evidence regarding its reproducibility and operational impact. Quantitative comparisons with alternative regulatory support models were not performed. In addition, hybrid decentralization within the described trials was limited to clinical laboratory assessments conducted at affiliated health system locations rather than fully decentralized trial participation.

## Future directions

Future work will evaluate application of the Sponsor-Proxy model across additional therapeutic areas and investigator-initiated studies. Ongoing evaluation will also assess impacts on investigator engagement, trial activation timelines, and participant access to clinical research. Continued development of institutional workforce capacity and operational infrastructure will support expansion of investigator-initiated trials and facilitate translation of emerging scientific discoveries into early-phase clinical investigation. Integration of EHR–based platforms (e.g., Epic), including embedded analytics and emerging AI-enabled functionalities, may further support Sponsor-Proxy operations by enhancing patient identification, data capture, and real-time safety monitoring while augmenting SSU staff efficiency.

## Figure 1. Long description

The figure depicts the translational pathway used for development of INM176. Preclinical discovery includes identification of the investigational product and animal pharmacokinetic and efficacy studies. Key barriers include botanical chemistry, manufacturing and controls complexity, sponsor regulatory burden, and rural access to clinical research. Sponsor-Proxy infrastructure consists of the Investigator-Initiated Trial Sponsor Support Unit and collaboration between PhD and physician investigators. Regulatory and trial development activities include Investigator’s Brochure preparation, protocol development, IND submission, and parallel regulatory and operational workflows. Clinical implementation includes IND authorization without clinical holds, hybrid decentralized laboratory workflows, and expansion of investigator-initiated translational programs.

Navigate back to Figure 1..

## Figure 2. Long description

The figure illustrates division of responsibilities within the Sponsor-Proxy model. The Scientific Sponsor provides scientific leadership, preclinical discovery, pharmacokinetic and pharmacodynamic biomarker development, and coordination of investigational product supply. The Sponsor Support Unit performs sponsor-level operational functions including IND preparation, regulatory management, protocol and Investigator’s Brochure development, monitoring plan development, monitor training, DSMC coordination, safety oversight, clinical data management, Trial Master File management, and regulatory compliance. The Clinical PI and Study Team conduct clinical trial activities including participant recruitment, clinical care, participant safety monitoring, clinical coordination, and data capture. Participants undergo trial participation and protocol-specified safety assessments.

Navigate back to Figure 2..

## Table 1. Long description

IND documentation requests included submission of an Investigator’s Brochure, which the Sponsor Support Unit developed using preclinical pharmacology, toxicology, and prior clinical data. Botanical product characterization requests involved clarification of extract composition and active compound content, addressed through manufacturer documentation and analytical verification of decursin content. Chemistry, Manufacturing, and Controls requests included manufacturing processes, technology transfer, and contaminant testing, addressed through integration of manufacturing records, certificates of analysis, and quality testing documentation. Clinical protocol safety design requests involved revisions to dose-limiting toxicity definitions, eligibility criteria, and safety monitoring procedures, addressed through protocol revisions and statistical design updates. Human subject protection requests involved expansion of informed consent language related to adverse events and reproductive risk, addressed through updates to informed consent documentation.

Navigate back to Table 1..

## Table 2. Long description

The table outlines division of responsibilities between the Sponsor Support Unit and the Clinical PI Study Team. For trial design, the Sponsor Support Unit is responsible for protocol development and Investigator’s Brochure preparation, while the Clinical PI Team provides clinical feasibility input. For regulatory oversight, the Sponsor Support Unit performs IND preparation, submission, and FDA communication, while the Clinical PI Team ensures site regulatory compliance. Documentation responsibilities include Trial Master File management by the Sponsor Support Unit and source documentation by the Clinical PI Team. Safety oversight includes sponsor safety reporting and DSMC coordination by the Sponsor Support Unit and clinical safety monitoring by the Clinical PI Team. Monitoring responsibilities include monitoring plan development by the Sponsor Support Unit and site coordination by the Clinical PI Team. Data management responsibilities include sponsor documentation oversight by the Sponsor Support Unit and data capture and entry by the Clinical PI Team. Participant management responsibilities remain with the Clinical PI Team and include recruitment, clinical care, and electronic health record monitoring.

Navigate back to Table 2..
